# The Role of Engagement in Teleneurorehabilitation: A Systematic Review

**DOI:** 10.3389/fneur.2020.00354

**Published:** 2020-05-06

**Authors:** Marta Matamala-Gomez, Marta Maisto, Jessica Isbely Montana, Petar Aleksandrov Mavrodiev, Francesca Baglio, Federica Rossetto, Fabrizia Mantovani, Giuseppe Riva, Olivia Realdon

**Affiliations:** ^1^“Riccardo Massa” Department of Human Sciences for Education, University of Milano-Bicocca, Milan, Italy; ^2^IRCCS Fondazione don Carlo Gnocchi ONLUS, Milan, Italy; ^3^Department of Psychology, Università Cattolica del Sacro Cuore, Milan, Italy; ^4^Applied Technology for Neuro-Psychology Laboratory, Istituto Auxologico Italiano, IRCCS, Milan, Italy

**Keywords:** engagement, self-management, patient activation, digital technologies, teleneurorehabilitation

## Abstract

The growing understanding of the importance of involving patients with neurological diseases in their healthcare routine either for at-home management of their chronic conditions or after the hospitalization period has opened the research for new rehabilitation strategies to enhance patient engagement in neurorehabilitation. In addition, the use of new digital technologies in the neurorehabilitation field enables the implementation of telerehabilitation systems such as virtual reality interventions, video games, web-based interventions, mobile applications, web-based or telephonic telecoach programs, in order to facilitate the relationship between clinicians and patients, and to motivate and activate patients to continue with the rehabilitation process at home. Here we present a systematic review that aims at reviewing the effectiveness of different engagement strategies and the different engagement assessments while using telerehabilitation systems in patients with neurological disorders. We used PICO's format to define the question of the review, and the systematic review protocol was designed following the Preferred Reported Items for Systematic Reviews and Meta-Analysis (PRISMA) guidelines. Bibliographical data was collected by using the following bibliographic databases: PubMed, EMBASE, Scopus, and Web of Science. Eighteen studies were included in this systematic review for full-text analyses. Overall, the reviewed studies using engagement strategies through telerehabilitation systems in patients with neurological disorders were mainly focused on patient self-management and self-awareness, patient motivation, and patient adherence subcomponents of engagement, that are involved in by the behavioral, cognitive, and emotional dimensions of engagement. Conclusion: The studies commented throughout this systematic review pave the way for the design of new telerehabilitation protocols, not only focusing on measuring quantitative or qualitative measures but measuring both of them through a mixed model intervention design (1). The future clinical studies with a mixed model design will provide more abundant data regarding the role of engagement in telerehabilitation, leading to a possibly greater understanding of its underlying components.

## Introduction

In the field of neurorehabilitation, one of the main objectives after a brain or nerve injury is to develop rehabilitation strategies directed at the recovery of functional skills by enhancing neuroplasticity (2). Even though the type of intervention, intensity, and number of sessions are known to be important in task-specific rehabilitation trainings (3), it is known that the role of engagement is key for enhancing neuroplasticity, and to facilitate functional recovery in patients with neurological disorders (2, 4). In this regard, some studies observed that by increasing patients' attention and interest toward rehabilitation training, there is an updating and modification at a neurological level, which leads to improving functional outcomes (5). However, to achieve such positive functional outcomes in neurorehabilitation, the nervous system has to be engaged and challenged (5, 6). From a neurobiological point of view, several studies have shown how engagement may increase neural activity in different cortical areas such as (2) the orbitofrontal regions, that integrate information from sensory and motivational pathways to generate pleasure, (3) the ventral striatal dopaminergic systems, and (4) the anterior cingulate cortex, which holds attention during demanding task execution (7). Even though there are not enough studies using neuroimaging techniques to demonstrate the effects of engagement in neuroplasticity for rehabilitation, a large amount of studies using mental practice techniques, enriched environments, and attentional and motivational strategies in which patients become active actors of the rehabilitation training, corroborates the relationship between engagement and neuroplasticity (8–10). Concerning this, the growing development of technology in the last decade lead to the introduction of new digital systems in rehabilitation through which it is possible to provide different sensory stimuli enhancing patients' resources such as attention and motivation. Thus, digital technologies in rehabilitation are directed to providing information and/or support emotional, behavioral, or physiological features of the pathology within an enriched and stimulating environment (11–14). One interesting feature of digital technologies in rehabilitation is the opportunity to apply technology-based interventions to provide a rehabilitation service through digital and telecommunication technologies during the hospitalization period, or at home after discharge from the hospital (15). Such application of digital technologies for rehabilitation is commonly known as telerehabilitation (16). Moreover, through telerehabilitation systems is possible to engage patients by providing them an online (or offline) feedback of their outcomes through a double communication loop (17, 18). This type of communication combines remote monitoring of patients' performance with clinicians' appropriate responses by adapting and personalizing the planned rehabilitation activities, and empowering patients toward the targeted rehabilitation aim (18, 19). Further, through these types of telerehabilitation systems, clinicians can supply the needs of the patients in long-lasting rehabilitation programs after the hospitalization period, allowing them to remain involved in social and productive life even though of their clinical condition (17). Moreover, through telerehabilitation systems clinicians have the possibility of delivering long rehabilitation trainings in an enriched digital environment at patients' homes while saving a big amount of sanitary costs (20). Thus, the use of telerehabilitation systems can enhance the patients' engagement by conducting their rehabilitation training at home. However, how to enhance engagement and what engagement is when using telerehabilitation systems in patients with neurological disorders is not clear enough. Due to this, the following section aims to clarify some components and subcomponents of engagement at a clinical level.

### Patient-Centered Medicine and Engagement

When we refer to patient engagement in the clinical field, we have to refer to patient-centered medicine (PCM). These two concepts are associated given that PCM considers a patients active participation in the clinical process as pivotal, instead of only considering the clinical professionals' point of view (21). In that context, patient engagement was considered as a concept to qualify the exchange between patients' demands and clinicians' supplies (22). Further, in healthcare, the term “engagement” came to indicate a renewed partnership between patients and healthcare providers (23). Then, the main goal of engaging patients in their clinical process can be identified in making them conscious of the management of their health status and illness, and to provide more positive outcomes in healthcare (24). Indeed, during the clinical process, patient engagement is a key factor in making them feel like participants in the therapeutic process that will lead to better adherence to the therapy, patient sensitization, and patient knowledge and empowerment (25). Even though the term “engagement” seems clear enough by itself, it involves different factors that have to take into consideration when engaging patients in a therapeutic process. Specifically, the involved factors in engagement are the following: participation and decision making, compliance and adherence, self-management, patient empowerment, and patient activation.

#### Participation and Decision Making

One of the main objectives for the improvement of the quality of health services defined by Entwistle and Watt (26) is the ability to involve patients in their therapeutic process by collaborating with the healthcare professionals. Two main factors have been defined for involving patients in clinical practices: patient participation and patient decision making. The first, patient participation, is considered a psychological component that focuses on identifying emotional and cognitive factors to enhance the active participation of the patients in clinical decision making (27). The second one is centered on the clinical and relational skills of the healthcare professionals in involving patients in clinical decisions (28, 29). Altogether, when referring to engagement in a clinical context, one intends to increase the communication between clinicians and patients to motivate patient participation throughout the clinical process. That means, giving the patients enough information about their illness to become more independents in their healthcare routine. Then, an engaged patient is a patient that can participate in the clinical decision making and healthcare routine, but also a patient able to actively participate in the global healthcare system promoting new forms of assistance, for example by using new technology systems (30).

#### Compliance and Adherence

Other factors embedded in patient engagement are “compliance” and “adherence” that refer to the adaptive behaviors of patients in following medical prescriptions or in following the healthcare routine (31). Although these two factors are often presented together, there are some differences between them. While “compliance” is related to patients' ability in adapting their life routine with a more passive/dependent attitude to the clinicians' indications (32), “adherence” is related with patients participation as an active actor in the communication exchange with the clinicians in which patients' and clinicians' plan together the patients care routine (33). Hence, the level of compliance and adherence to the clinical process depend on patients' attitudes and behaviors in accepting or disagreeing with the clinicians' prescriptions, moving the concept of patients' engagement toward a balance between patients' demands and clinicians' supplies (30).

#### Self-Management, Patient Empowerment, and Activation

Self-management is referred to as the patients' ability to manage symptoms, treatments, psychological, and psychosocial consequences of their pathological condition, as well as the ability to manage the cognitive, behavioral, and emotional responses, derived from their clinical condition, to reach a satisfactory quality of life (34, 35). Indeed, self-management is considered a positive outcome of patient engagement during the clinical process. Moreover, patient empowerment is also considered an important positive outcome during the patient engagement process. It is known that the term “empowerment” refers to psychological resources through which patients can control their clinical condition and the related treatments (36, 37). Thus, by providing the patients an educational healthcare process, they can recover agency and beliefs of self-efficacy over their health condition increasing their autonomy at the same time (38). Even though the concept of “empowerment” and the concept of “engagement” are strongly related, “empowerment” is considered an outcome of a mainly cognitive boosting process of patients, related to their knowledge of the clinical condition, while “engagement” also sustains the emotional aspects regarding to the acceptance of the patients clinical conditions and the behavioral skills to manage it (30). Finally, patient activation is related to the capacity of the patients in managing their clinical condition and the ability to interact with the healthcare system based on their level of knowledge (39, 40). It is suggested that an increase in patient activation leads to an increase in healthy behaviors and adherence to the clinical process (23). Patient activation has been defined by Hibbard et al. (23) as composed of four phases: (1) the passive activation level, where patients are not aware of their role in their health management; (2) where patients starts to create their resources and knowledge about their health condition; (3) where patients can elaborate *ad hoc* responses to the problems related to their clinical condition; and (4) where patients can maintain their new lifestyle behaviors for long-term periods, even when they are under stressful situations. Then, following the later commented phases, Hibbard et al. created the patient activation measure (PAM) to assess patient activation (23).

Hence, patient engagement considers not only the clinical environment but also the non-clinical contexts such as patients' daily routines, activity routines, and the acceptance of their clinical condition outside the hospital, by exploring the dialogue between the supplies and demands of the healthcare services (41). Concerning this, the use of new digital technologies to achieve the patients' engagement during and after the hospitalization period has been proposed (42).

### Technology for Patient's Engagement in Neurorehabilitation

Today the development of new technologies has paved the way for their use for clinical purposes, especially to enhance patients' engagement in their healthcare routine (43). Recently, it has been demonstrated that the use of new digital technologies can modulate the dimensions described by Seligman (44) for positive psychology. Digital technologies have been considered essential for illness prevention such as courage, future-mindedness, optimism, interpersonal skill, faith, work ethic, hope, perseverance, flow, and joy (42). In this regard, it is known that the use of virtual environments and serious games can induce positive emotional states, creating new virtual environments for human psychological growth and well-being (45). Following the model proposed by Frome (46), four factors have to be present to induce positive emotions by using such virtual or serious games: a narrative factor, by using roleplaying through which is possible to feel the emotions of the virtual character; game-playing factor, by providing the feeling of frustration or satisfaction when winning or losing the game; the simulation factor, meaning that the game has to provide engaging activities; and the aesthetics factor, referring to the artistic features of the game. These factors can promote engagement of the users by using different technological sources such as mobile e-health (47), and e-learning platforms (48), biofeedback systems (49), virtual reality systems (50, 51), and playing videogames (45), at their own home.

In addition, new rehabilitation protocols, including the use of new technologies, have been developed in the neurorehabilitation field (52, 53). Particularly, the use of new technologies in neurorehabilitation, such as telerehabilitation systems, allows the patients to continue with their healthcare process at home (19, 54). In the field of neurorehabilitation, the rehabilitation and healthcare routine after the hospitalization period is complex, requiring a multidisciplinary coordination (55, 56). Telerehabilitation systems in neurorehabilitation allow a large number of people with neurological disorders—who often have limitations due to limited mobility and to costs associated with travel—to continue with their healthcare process at their own home, minimizing the barriers of distance, time and costs, and receiving continued support by the clinicians remotely (57, 58). The feasibility and efficacy of telerehabilitation systems in neurorehabilitation have been documented in patients with different neurological conditions such as patients in a post-stroke phase (59–61), Parkinson Disease (18, 62, 63), and Multiple Sclerosis (18, 64). Nevertheless, the role of engagement and the different factors to engage patients with neurological disorders in the telerehabilitation training during the rehabilitation period have not yet been deeply investigated. Hence, this systematic review aims at reviewing the effectiveness of different engagement strategies and the different engagement assessments while using telerehabilitation systems in patients with neurological disorders.

## Methods

A systematic review of the scientific literature have been conducted in order to identify different engagement strategies, as well as studies reporting engagement assessment methods when using telerehabilitation systems in patients with neurological disorders. The systematic review protocol was designed following the Preferred Reported Items for Systematic Reviews and Meta-Analysis (PRISMA) guidelines (65).

### Data Sources and Search Strategy

According to the PICO format to formulate the foreground question of this systematic review (66), the review question has been defined as, “in adults with neurological disorders, is the role of engagement for telerehabilitation interventions, compared to treatment as usual, effective in improving neurorehabilitation intervention.” Bibliographical data was collected on July 4, 2019, by using the following bibliographic databases: PubMed, EMBASE, Scopus, and Web of Science. For each database, we used the following combination of research keywords: (1) (“engagement” OR “motivation” OR “activation” AND “telerehabilitation”); (2) (“engagement” OR “motivation” OR “activation” AND “telehealth”); (3) (“engagement” OR “motivation” OR “activation” AND “telemedicine”); (4) (“engagement” OR “motivation” OR “activation” AND “telecare”). See the detailed search strategy in Table 1. Only full-text available articles were included in our research (conference paper were excluded), studies citation were retrieved independently for each string of keywords across all databases. Finally, the first list of the collected studies during the bibliographic research was exported to Mendeley to remove duplicated studies. Then the list of studies without duplicates was imported to Rayyan (67) for the title and abstract screening, following the specified inclusion or exclusion criteria for study selection (see section Study Selection and Data Collection) by one reviewer (M.M.G). The final list of the selected studies was sent to leading experts in the field for suggestion and identification of any missing studies, and no studies were added.

**Table 1:** Data search strategy.

**“Engagement” OR “motivation” OR “activation” AND**
	**PubMed**	**EMBASE**	**Scopus**	**Web of science**	
	**Abs/Tit**	**Article**	**Article**	**Article**	**Total_keyword**
Telerehabiliation	41	52	275	59	427
Telehealth	216	1115	967	271	2569
Telemedicine	293	821	2461	391	3966
Telecare	32	67	854	38	991
Total	582	2055	4557	759	7953
Total to analyze without duplicates					4618

### Study Eligibility Criteria

The present review aims at reviewing the effectiveness of different engagement strategies and the different engagement assessments while using telerehabilitation systems in patients with neurological disorders. Then, the selected studies had to investigate engagement while using telerehabilitation systems in adult patients with neurological disorders. Bibliographical research was limited to studies using humans and written in English. Further, the selected studies had to accomplish the following inclusion criteria:

1. Telerehabilitation interventions must have been directed to engage patients in their healthcare routine. Interventions directed to engage other stakeholders such as medical staff, hospital managers, and others were excluded.
2. Telerehabilitation interventions must have been directed to a group of patients, with a between or within-group study design. Single case studies have been excluded.
3. Telerehabilitation interventions have been directed to assess one or more components of patient engagement.

### Study Selection and Data Collection

One reviewer (M.M.G.) conducted the final selection of the studies for full text analyses. The following keywords were considered as inclusion criteria for selected articles in Rayyan (67): neurorehabilitation, neurological patients, patients, participation, adherence, self-management, empowerment, activation, telerehabilitation, telehealth, telemedicine, telecare, e-health. Further the following keywords were considered as exclusion criteria: no engagement, no neurological patients, animal studies, and review studies. Then, the final selected articles that accomplished the inclusion criteria were analyzed by three reviewers (M.M.G., M.M., and J.M.) for independently full-text analyses. The final selected studies were discussed among the three reviewers in order to solve minor discrepancies about the study selection criteria that had been solved by consensus.

### Risk of Bias Assessment

To the risk of bias assessment, the reviewers followed the guideline of the Cochrane Collaboration risk of bias tool according to the latest version of the risk of bias tool (RoB2) statement (68). All three reviewers (M.M.G, M.M, and J.M) independently evaluated the studies for risk of bias, and disagreements were resolved through consensus (Table 2).

**Table 2:** Risk of bias assessment.

**References**	**Random sequence generation (selection bias)**	**Allocation concealment (selection bias)**	**Blinding of participants and personnel (performance bias)**	**Blinding of outcome assessment (detection bias): self-reported outcomes**	**Incomplete outcome data (attrition bias)**	**Selective reporting (reporting bias)**	**Other bias**
Yeh et al. (69)	High	High	High	Low	Low	Low	High: small smaple size/no control group/no homogeneous clinical sample
Llorèns et al. (70)	High	High	High	Low	Low	Low	High: small sample size/no control group
White et al. (71)	High	High	High	High	Low	Low	High: small sample size/no control group/only interview assessment
Ferreira et al. (72)	High	High	High	Low	Low	Low	High: small sample size
Nijenhuis et al. (73)	High	High	High	Low	Low	Low	High: small sample size/no control group
Lloréns et al. (74)	Low	Low	Low	Low	Low	Low	Low
Palacios-Ceña et al. (75)	High	High	High	High	Low	Low	High: small sample size/no control group/only interview assessment
Houlihan et al. (76)	Low	Low	Low	Low	Low	Low	Low
Engelhard et al. (77)	High	High	High	Low	Low	Low	High: no control group
Lai et al. (78)	High	High	High	Low	Low	Low	Low
Skolasky et al. (79)	Low	Low	Low	Low	Low	Low	Low
Pitt et al. (80)	High	High	High	Low	High	Low	High: small sample size/no control group
D'hooghe et al. (81)	High	High	High	Low	Low	Low	High: no control group
Dennett et al. (82)	Low	Low	Low	Low	Low	Low	Low
De Vries et al. (83)	High	High	High	High	Low	Low	High: small sample size/no control group/only interview assessment
Thomas et al. (84)	High	High	High	High	Low	Low	High: small sample size/no control group/only interview assessment/no homogeneous clinical sample
Chemtob et al. (85)	High	High	High	High	Low	Low	High: small sample size/only interview assessment
Ellis et al. (86)	Low	Low	Low	Low	Low	Low	Low

*High, High risk of bias; Low, Low risk of bias*.

### Data Extraction

Each selected study was coded according to the following thematic categories: (1) Authors and Year of publication; (2) Clinical condition (N); (3) Patients characteristics; (4) Sample size; (5) Control group; (6) Type of engagement; (7) Engagement assessment; (8) Main results (Table 3). All three reviewers followed the coding studies criteria to analyze the final selected studies. Further, the TiDER checklist has been used for reporting detailed information about research interventions (87). Specifically, the following points of the TiDER checklist have been reported: (1) why (aim of the study), (2) what (materials), (3) who provided, (4) tailoring, and (5) intervention adherence (Table 4).

**Table 3:** Overall studies characteristics.

**References**	**Clinical condition [total sample size]**	**Patients characteristiscs**	**Case vs. control group [size]**	**Control group [type]**	**Case group [type of engagement]**	**Engagement assessment**	**Main results**
Yeh et al. (69)	Stroke, TBI, SCI [*N =* 14]	Unspecified	[14 vs. –]	No	Emotional engagement (secondary outcome of the study)	The mood was measured with the POMS questionnaire; experience of “presence” in the telerehabilitation environment, willingness to persist with therapy, and a telerehabilitation usability questionnaire	Patients felt less efficacious in continuing therapy after participating in the telerehabilitation game compared to their reported perseverance self-efficacy before the game and showed a decreased willingness to persist in therapy regardless of fatigue after the gameplay.Telerehabilitation significantly enhanced stroke patients' psychological states
Lloréns et al. (70)	ABI [*N =* 10]	Chronic phase (> 6 months)	[10 vs. –]	No	Self-awareness game, that consist in answering questions related to knowdledge (anatomical and pathological matters), reasoning (situational exercises), action (role-playing), or cohesion (jokes and sayings), in a competitive context	Self-Awareness Deficits Interview (SADI) Social Skills Scale (SSS)	The VR game improved self-awareness and the social cognition deficits in patients with ABI after the 8 months training period
White et al. (71)	Stroke [*N =* 12]	Unspecified	[12 vs. –]	No	Face-to-face sessions aimed to provide orientation to the iPad, educate toward therapist recommended rehabilitation Apps and access to other tablet technology features	Telephonic semi-structured interviews	Stroke survivors experienced increased participation in therapeutic activities, increased socialization, and less inactivity and boredom
Ferreira et al. (72)	PD [*N =* 33]	Mild-to-moderate stage (Hoehn and Yahr score 1–2.5)	[22 vs. 11]	Usual care	Biofeedback from the system and weekly telephonic interviews	Semi-structured interviews to assess willingness to continue in the study, satisfaction with the SENSE-PARK System, changes in health status or medical condition, adverse events, feedback messages, and doubts about the system	Motivation to wear such a system can be increased by providing direct feedback about the individual health condition
Nijenhuis et al. (73)	Stroke [*N =* 24]	Chronic phase (> 6 months)	[24 vs. –]	No	Video-game and remote supervision of the clinicians	Intrinsic Motivation Inventory (IMI)	Participants were able and motivated to use the training system independently at home. Usability shows potential, although several usability issues need further attention
Lloréns et al. (74)	Stroke [*N =* 45]	Chronic phase (> 6 months)	[30 vs. 15]	Training at the hospital.	Engagement as a secondary outcome	Usability Scale (SUS) Intrinsic Motivation Inventory (IMI)	Both groups considered the VR system similarly usable and motivating
Palacios-Ceña et al. (75)	MS [*N =* 24]	Unspecified	[24 vs. –]	No	Video-game and tracked movement feedback	Unstructured interviews	Four main themes emerged from the data: 1) regaining previous capacity and abilities. 2) Sharing the disease, 3) adapting to the new treatment. This refers to the appearance of factors that motivate the patient during KVHEP
Houlihan et al. (76)	SCI [*N =* 126]	Traumatic SCI, chronic phase (≥1year postinjury)	[84 vs. 42]	Usual care	Peer health coach (PHC), who acts as a supporter, role model, and advisor	Patient Activation Measure (PAM)	Intervention participants reported a significantly greater change in PAM scores compared with controls. Participants reported a significantly greater decrease in social/role activity limitations, greater services/resources awareness, greater overall service use, and a greater number of services used
Engelhard et al. (77)	MS [*N =* 31]	MS with Expanded Disability Status Scale ≤ 6.5	[31 vs. –]	No	A dedicated “Symptom Tracker” page allowed subjects to compare severity between symptoms and view recent trends	Completion of the web-exercises	52% of the subjects reported improved understanding of their disease, and approximately 16% wanted individualized wbPRO content. Over half of perceived well-being variance was explained by MS symptoms, notably depression, fatigue, and pain
Lai et al. (78)	PD [*N =* 30]	Mild-to-moderate stage (Hoehn and Yahr score 1–3)	[20 vs. 10]	Self-regulated exercises	To instruct participants on proper exercise techniques to increase mastery, discuss barriers or issues with the participants' ability to attend the exercise sessions, help participants set achievable goals to complete the exercise prescription, provide verbal encouragement to achieve the desired exercise workload	Measures of adherence included four variables: number of sessions performed, time of exercise, and attendance	Internet supervised training at home could promote stronger program adherence than self-managed home-exercise training. The telehealth system, telecoaches provided a sense of companionship and accountability and bolstered participants' confidence to overcome several impediments to participation
Skolasky et al. (79)	LSS [*N =* 182]	post-surgery phase	[122 vs. 60]	Usual care	Telephone-based intervention engagement	Engagement is a secondary outcome	Health behavior change counseling improved health outcomes after the surgical procedure through changes in rehabilitation engagement
Pitt et al. (80)	Aphasia [*N =* 19]	Unspecified	[19 vs. –]	No	Video-conferences to create opportunities for communicative success, to share personal life history, and to provide support for living successfully with aphasia through networking with others	Quality of Communication Life Scale. Communicative Activities Checklist Engagement a secondary outcome	Improvements in communication-related quality of life increased engagement in communicative activities and decreased aphasia severity
D'hooghe et al. (81)	MS [*N =* 57]	Relapsing-remitting MS with Expanded Disability Status Scale ≤ 4	[57 vs. –]	No	A combination of self-management and motivational messages, to enhance self-energy management and physical activity to improve the level of fatigue in pwMS	Modified Fatigue Impact Scale (MFIS) Short Form-36 (SF-36) Hospital Anxiety Depression Scale (HADS)	MS TeleCoach is a potential self-management tool to increase activity and reduce fatigue
Dennett et al. (82)	MS [*N =* 135]	Unspecified	[90 vs. 45]	Conventional home (paper format)	Web-based exercises with personal conversational support through the weekly interviews	Interviews	The web-based physio is important for building in conversations with people with MS about expectations of exercise and its potential benefits, particularly for those whose condition is deteriorating
Vries et al. (83)	PD [*N =* 16]	Unspecified	[16 vs. –]	No	Video recorded movement observation.	Semi-structured interviews after the software exposure	The following conditions were identified to foster patients' engagement: Camera recording (e.g. being able to turn off the camera), privacy protection (e.g. patients' behavior, patients' consent, camera location) and perceived motivation (e.g. contributing to science or clinical practice)
Thomas et al. (84)	MS [*N =* 15]	Unspecified	[15 vs. –]	No	Telephonic interviews	Interviews	Particularly of interest were themes related to replicating the group dynamics and the lack of high-quality solutions that would support the FACETS' weekly homework tasks and symptom monitoring and management
Chemtob et al. (85)	SCI [*N =* 33]	SCI with paraplegia, chronic phase (≥1year postinjury)	[22 vs. 11]	Usual care	The counseling sessions focused on fostering the basic psychological needs and autonomous motivation, teaching behavior change techniques, and self-regulatory strategies	Conversation analyses	The intervention group reported greater autonomous motivation post-intervention. Large to moderate effects supporting the intervention group were found for health participation, and meaningful life experiences and social cognitive predictors. A trained physical activity counselor can increase physical activity motivation
Ellis et al. (86)	PD [*N =* 61]	Mild-to-moderate stage (Hoehn and Yahr score 1–3)	[44 vs. 21]	Active control group	Cognitive-behavioral elements to enhance the basic behavioral change component of the individualized exercise and walking program and to emphasize participants' engagement in managing their health condition	Daily records of steps taken and exercises performed, using either the mobile health application (mHealth group) or paper calendars (active control group)	Adherence to the exercise program was similar between groups. The addition of enhanced, remotely monitored, mobile technology-based, behavioral change elements to the exercise prescription appeared to benefit participants who were less active differentially

*TBI, Traumatic Brain Injury; ABI, Acquired Brain Injury; SCI, Spinal Cord Injury; MS, Multiple Sclerosis; PD, Parkinson disease; LSS, Lumbar spinal stenosis*.

**Table 4:** TiDER checklist study characteristics.

**References**	**Brief name**	**Aim**	**Set-up**	**Task**	**Who provided**	**How**	**Where**	**When/How much**	**Tailored**	**Intervention adherence**
Yeh et al. (69)	Motivation and Telerehabilitation	To provide a telerehabilitation experience to create an elevated mood state allowing patients and therapists to experience a sense of co-presence that will be associated with satisfaction with the telerehabilitation system, and willingness to persist in therapy	A telerehabilitation system composed of two subsystems: a motor rehabilitation system, and a tele-communication system	The therapists had to guide the patient through the setup of the systems and then talk him/her through three computer games designed to provide motor rehabilitation exercises for the upper extremity	Therapist (Unspecified role)	Remotely from placed at a different location through the telerehabilitation system	Therapist/patient pairs were taken into separate rooms.	Daily therapy during an unspecified time	The difficulty levels and the progress in gameplay were monitored and manipulated through a live video chat during the exercise	Two 7-point scale items measured daily therapy during an unspecified time the willingness to persist in therapy
Lloréns et al. (70)	Virtual reality for self-awareness	To study the effectiveness of the virtual system in the rehabilitation of self-awareness skills	A multi-touch non-immersive virtual reality system	Patients had to move forward in the virtual game by answering questions, which can be related to knowledge (anatomical and pathological matters, red cards), reasoning (situational exercises, blue cards), action (role-playing exercises, green cards), or cohesion (jokes and sayings, yellow cards), related to their clinical condition	Self-provided by the patients	Self-provided by the patients at hospital	At hospital	1-hour session per week during 8 months	No	No
White et al. (71)	Tablet acceptability in stroke survivors'	To explore stroke survivor acceptability of and experience of tablet use during the first three months of stroke recovery	Tablet technology	A qualitative study using an inductive thematic approach incorporating the process of constant comparison was utilized to collect and analyze data	Self-provided by the patients	Remotely	Patients' home	During the first three months of stroke recovery	Not specified	Qualitative outcomes were participants' perceptions using in-depth, semi-structured interviews
Ferreira et al. (72)	Teleassessement in pwPD	To assess the feasibility and usability of an objective, continuous, and relatively unobtrusive system (SENSE-PARK System	SENSE-PARK System which consists of wearable sensors, a smartphone-based App, a balance board, and computer software	To perform a balance and cognitive training	Two trained researchers were involved. The training was administered by the SENSE-PARK System	Remotely	Patients' home	Sensors' information was registered 24 hours/7 days over 12 weeks	Not specified	Semi-structured interviews were conducted by phone to gain insight into the experiences of the participants using the SENSE-PARK System. Topics discussed were: willingness to continue in the study, satisfaction with the SENSE-PARK System, changes in health status or medical condition, adverse events, feedback messages, and doubts about the system
Nijenhuis et al. (73)	A motivational self-administered training for stroke	To assess the feasibility and potential clinical changes associated with a technology-supported arm and hand training system at home for patients with chronic stroke	A computer containing user interface and games, Touchscreen and SaeboMAS, SCRIPT wrist and hand orthosis	To perform an upper limb training combining assisted movement by an orthosis and motor videogame	Trained clinical researchers (human movement scientists), physical therapists, or occupational therapists remotely	Remotely	Patients' home	30 minutes of exercise per day, 6 days per week	Game difficulty schedule was used by the HCP weekly to provide the correct game categories to each participant. The HCP adjusted the training program remotely by accessing the HCP user interface	The System Usability Scale is a 10-item scale to assess a global view of the subjective experience of system usability
Lloréns et al. (74)	Telerehabilitation of balance after stroke	To evaluate the clinical effectiveness of a virtual reality-based telerehabilitation program in recovering balance compared to an in-clinic program in hemiparetic patients with stroke. Second, to compare the subjective experiences, and finally, to contrast the costs	The hardware system consisted of a TV, a standard computer, and a Kinect™ (Microsoft®, WA). A 42” LCD screen and a PC were used in the clinical setting	The VE used in the experiment represented the participants' feet and their movements in an empty scenario, which consisted of a checkered floor that facilitated the depth perception, with a central circle that represented the center of the VE. Different items rose from the floor around the circle	Two physical therapists were involved remotely to detect possible issues and act accordingly	Remotely	Patients' home	45-minute training sessions, 3 days a week, during 8 weeks.	The level of difficulty of the task was defined by configuring the region of appearance, distance, size, lifetime, and number of simultaneous items. The difficulty of the task was adjusted automatically by the system	The System Usability Scale is a 10-item scale to assess a global view of the subjective experience of system usability
Palacios-Ceña et al. (75)	Kinect VR home-based program in pwMS	To explore the experiences of multiple sclerosis patients who performed a virtual home-exercise program using Kinect	Kinect home-exercise program	Postural control and balance exercises	Medical doctors and therapists were involved in the recruitment and assessment times	Remotely	Patients' home	10-week training	Unspecified	Unstructured interviews, using open questions, and thematic analysis were conducted
Houlihan et al. (76)	Enhancing self-management in pwSCI	To evaluate the impact of “My Care My Call” (MCMC), a peer-led, telephone-based health self-management intervention in adults with chronic spinal cord injury (SCI)	Telephone	Trained peer health coaches applied the person-centered health self-management intervention	Trained peer health coaches	Remotely	Patient's home	6 months on a tapered call schedule	Unspecified	Phone interviews
Engelhard et al. (77)	Remotely engagement in MS	To evaluate web-based patient-reported outcome (wbPRO) collection in pwMS in terms of feasibility, reliability, adherence, and subject-perceived benefits; and quantify the impact of MS-related symptoms on perceived well-being	Web portal	Patients had to report symptoms from home and view their symptom history. Subjects were required to complete each of the five questionnaires	Unspecified	Remotely	Patients' home	One per month during 6 months	No	Questionnaires at the web portal
D'hooghe et al. (81)	MS Telecoach feasibility	To enhance levels of physical activity, thereby improving fatigue in pwMS in an accessible and interactive way, reinforcing self-management of patients	Smartphone application consisting of two main components: telemonitoring and telecoaching	Patients had to perform a physical activity training while they were telemonitored and telecoached	Unspecified	Remotely	Patient's home	2- week run-in period was followed by a 12-week evaluation period	No	Telemonitored information about physical activity by the smartphone application. Visual analogue scale to assess levels of fatigue
Lai et al. (78)	Telemonitored rehabilitation in pwPD	To explore the uptake and implementation of Tele-Monitored Home-Exercise program in adults with PD	Android computer tablet with Bluetooth and wireless Internet capability, mounted to an adjustable floor stand. A wearable physiologic monitor (BioHarness 3, Zephyr)	Combined strength and aerobic exercise. Participants exercised under a telecoachs' supervision via videoconferencing	Research staff	Remotely	Patients' home	8 weeks of exercise, 3 sessions per week: with a total of 24 sessions	No	Measures of adherence included four variables: (a) the total number of exercise sessions performed, (b) time in minutes exercising per week, (c) time exercising at a moderate aerobic intensity per week, and (d) attendance. Interviews included 10 open-ended questions that served as general prompts for discussion in the following areas: perceptions of the program, equipment/devices, exercise setting, telecoach (or not having one), and rationales for exercise adherence
Skolasky et al. (79)	Improving Rehabilitation Engagement After Spinal Stenosis Surgery	To compare the effectiveness of health behavior change counseling with usual care to improve health outcomes after lumbar spine surgical procedures	Telephone	Health behavior change counseling is a brief, telephone-based intervention intended to increase rehabilitation engagement through motivational interviewing strategies that elicit and strengthen motivation for change	Clinical staff	Remotely	Patients' home	Participants were assessed before the surgical procedure and for 3 years after the surgical procedure for pain intensity	No	Phone interviews
Pitt et al. (80)	Telerehabilitation in pw aphasia	To describe changes in aphasia severity, and communication-related QOL and participation, for people with chronic aphasia following TeleGAIN	Web-based videoconferencing	Treatment provided opportunities to participate in a conversation, engage with others with aphasia, and complete functional communication activities	Clinicians and patients	Remotely	Patients' home	12 weeks	No	Communication-related quality of life and participation assessments
Dennett et al. (82)	Web-based physical intervention in pwMS	To explore the experiences of participants who used a web-based physiotherapy intervention as part of a feasibility randomized controlled trial by in-depth interviews	Web-based exercise platform	Patients had to perform a web- based exercise program	Physical therapist	Remotely	Patients' home	Twice-weekly web-based physiotherapy sessions.	No	Interviews were audio-recorded, transcribed verbatim, and analyzed using thematic analysis
Vries et al. (83)	Home-based video intervention in pwPD	To study the barriers and facilitators as perceived by PD patients considering continuous video recording at home for medical research and/or medical treatment purposes	Home-based video system + Kinect camera, which measures motor functioning	Patients had to perform their motor training routine, and it was recorded through the Kinect to the assessment of movement parameters, including standing up and several gait parameters	Research staff	Remotely	Patients' home	Motor training: not specified Interviews were conducted during 1 year	No	Interviews were semi-structured and included a standardized introduction, open-ended questions, and prompts to encourage further discussion and more specific answers
Thomas et al. (84)	Digital fatigue management in pwMS	To gather views about a web-based model of service delivery from HCPs who had delivered FACETS and from pwMS who had attended FACETS	Telephone	Telephone consultations were undertaken with FACETS-trained HCPs who had the experience of delivering FACETS	Clinicians	Remotely	Patients' home	Face to face consultation intervention	No	Interviews
Chemtob et al. (85)	Telehealth to enhance motivation in pwSCI	To test a pilot tele-health intervention, grounded in self-determination theory, to enhance need satisfaction, motivation, physical activity, and quality of life among adults with SCI.	Online video-chat platform.	Patients had to perform a leisure-time physical activity program that has been supported by an online coach intervention	Psychologist	Remotely	Patients' home	The Intervention group received online 1hour of counseling session per week, during 8 weeks	No	Online counseling
Ellis et al. (86)	Effectiveness of mHealth in pwPD	To explore the preliminary effectiveness, safety, and acceptability of a mobile health (mHealth)–a mediated exercise program designed to promote sustained physical activity in people with PD	Mobile health (mHealth)	Patients had to perform a mobile health–mediated exercise program (“mHealth” condition) with an exercise program administered without mobile health technology	Unspecified	Remotely	Patients' home	12-month single-blind (assessor)	No	Exercise adherence data were collected via daily records of steps taken and exercises performed, using either the mobile health application. Program acceptability was assessed after 12 months by having participants rate their satisfaction using a 1 to 10 Likert scale

## Results

### Study Selection

**Figure 1:**
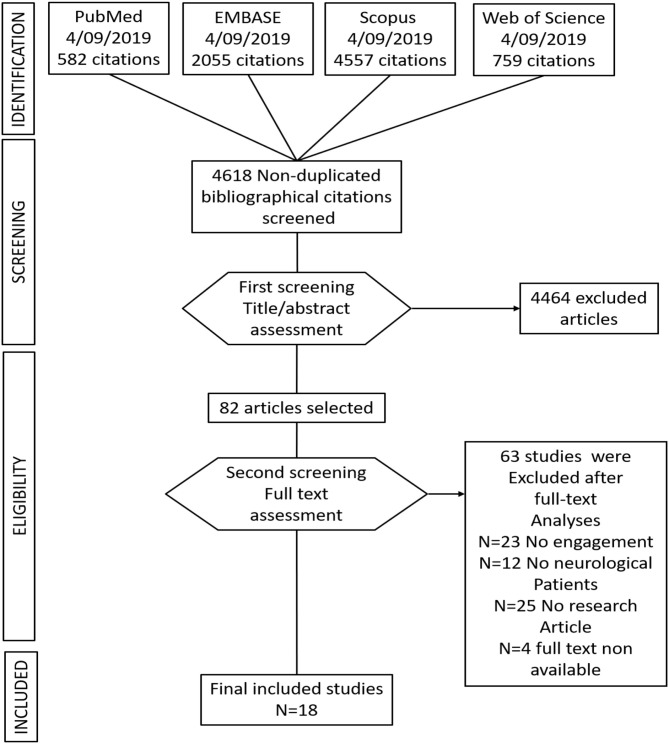
Flow chart of the study selection.

Seven thousand nine hundred and fifty three studies were found, including the above commented key words in section Data Sources and Search Strategy, and including the above-specified inclusion criteria words (section Study Selection and Data Collection). After removing duplicate studies, a total of 4,618 studies were included for the title and abstract screening into the Rayyan software. Of 4,618 non-duplicate studies, 4,464 studies did not accomplish the described study eligibility criteria. Subsequently, 82 studies were selected for full-text analyses. Of the 82 full text analyzed studies, only 18 studies were identified as suitable with the above-described inclusion criteria. See Figure 1 for a flow diagram depicting the study selection process.

Of 82 studies, only 18 studies included engagement strategies and engagement assessment either as a primary or secondary outcome after the telerehabilitation training in patients with neurological disorders.

### Study Characteristics

The final eighteen selected studies were described in detail. Further, Table 3 shows the characteristics of each of the selected studies. Ten studies compared patients with neurological disorders with healthy subjects or with other group of patients (69, 72, 74, 76–79, 82, 85, 86). Among the selected studies four studies were conducted in patients with Parkinson Disease (PD) (72, 78, 83, 86), four in patients with stroke (69, 71, 73, 74), and five studies were conducted in patients with multiple sclerosis (MS) (75, 77, 81, 82, 84). All the selected studies used engagement strategies in their telerehabilitation program, as well as engagement assessment measures. Particularly, eight studies used interviews to obtain qualitative data of patient engagement (69, 71, 74, 75, 82–85), six studies used functional assessment scales (70, 72, 73, 76, 80, 81), and three studies used paper or digital diary reports (77, 78, 86).

Moreover, following the TiDER checklist for reporting research interventions (87), the following points have been reported in Table 4: (1) why (aim of the study), (2) what (materials), (3) who provided, (5) tailoring, and (6) intervention adherence. (2) Out of the eighteen analyzed studies, thirteen studies aimed at investigating the effectiveness, usability, feasibility, reliability, and acceptability of the telerehabilitation system (70–75, 77–79, 82–84, 86), one study aimed at investigating the sense of co-presence between the therapist and patients through the telerehabilitation system (69), three studies aimed at investigating changes in self-management, self-determination, and self-motivation after the telerehabilitation period (76, 81, 86), and finally one study aimed at assessing possible changes in aphasia severity after the telerehabilitation period (80). (3) Five studies used a computer-based telerehabilitation system (69, 73–75); three studies used a tablet set-up as a telerehabilitation platform (70, 71, 78); three studies used patients smart phones applications for psychological or motor telerehabilitation programs (72, 81, 86); three studies used phones as a set-up for telephone-based telerehabilitation intervention (76, 79, 82); finally, three studies used an online web-platform as an internet-based telerehabilitation intervention (77, 80, 85). (4) Out of the 18 selected studies, nine studies involved therapists (physiotherapist, psychologist, medical, coach therapist) or medical doctors in the administration of the telerehabilitation program (69, 74–76, 79, 80, 82, 84, 85); four studies involved trained researchers in the administration of the telerehabilitation program (72, 73, 78, 83), two studies described a patients self-administered telerehabilitation program (70, 71), and three studies did not specify who was involved into the telerehabilitation program (77, 81, 86). (5) Out of the 18 analyzed studies, only three studies adjusted the difficulty levels of the telerehabilitation program automatically according to the progress of the patients among the rehabilitation period (69, 73, 74). (6) Out of the 18 analyzed studies, only one study did not assess adherence to the intervention (70). Among the other 17 studies, 11 studies used semi-structured or unstructured interviews to assess patients adherence to the telerehabilitation program (71, 72, 75, 76, 78–84). Four studies used questionnaires (74, 75, 77, 86), two studies used the assessment report collected from the mobile or tablet rehabilitation application (78, 86), and one study used the online counseling feedback to assess patients adherence to the telerehabilitation program (85). In addition to the latter commented points, Table 4 shows more detailed information about the research intervention of each study.

### Risk of Bias

All studies except five presented a high risk of bias in some of the assessed factors in this systematic review (74, 76, 79, 82, 86). Table 2 shows the results of the risk of bias assessment of this systematic review. All the studies included in this systematic review reported the sampling method. However, only five out of 18 studies presented a randomized control trial study design, including a control group for treatment comparisons (74, 76, 79, 82, 86). Ten studies presented an small sample size to represent the results obtained after the treatment period (69–73, 75, 80, 83–85). Five studies based their results on the analyses of interviews conducted to the patients without analyzing any other clinical measure for engagement assessment (71, 75, 83–85). All the studies included in this review reported their allocation sample method and study design. However, 12 studies did not have used random allocation methods for the sample allocation and not included a control group in the study design (70).

### Engagement Interventions in Teleneurorehabilitation

Once the final 18 studies included in this systematic review have been analyzed, the studies were divided in those in which engagement was considered a primary outcome of the telerehabilitation training (*n* = 11) (70–72, 76–79, 81, 82, 84, 85), and those in which engagement was considered a secondary outcome of the telerehabilitation training (*n* = 7) (69, 73–75, 80, 83, 86).

#### Engagement as a Primary Outcome

Most of the 11 analyzed studies aimed at investigating the patient engagement as a primary outcome through a telerehabilitation training in patients with neurological disorders. In specific those studies involving patients' self-management, self-awareness, and self-determination strategies to enhance active patients' participation in their healthcare routine, and providing patients' empowerment. Such engagement strategies have been included in the behavioral and cognitive dimension of engagement (88). Specifically, in the present systematic review, four studies directed to enhance the behavioral and cognitive dimension of engagement while using telerehabilitation systems have been found. For instance, a non-immersive virtual reality multitouch system had been used in 10 acquired brain injury patients (ABI) at home to treat self-awareness deficit (70). Particularly, patients were engaged in a self-awareness game consisting of answering questions related to knowledge (anatomical and pathological matters), reasoning (situational exercises), action (role-playing), or cohesion (jokes and sayings), in a competitive context (70). Further, in another study, the authors used a smartphone application for both the telemonitoring and tele-coaching of 57 patients with multiple sclerosis (MS) (81). The study by D'hooghe et al. aimed at fostering patients' self-energy management and physical activity, decreasing the level of fatigue after physical activity. Regarding patients with MS, a web-based model (FACETS: Fatigue: Applying Cognitive-behavioral and Energy effectiveness Techniques to life Style) of service delivery from healthcare providers was also tested in 15 patients with MS to improve patients' behavioral and cognitive dimension of engagement (84). Further, an online video-chat platform was used as a pilot test telehealth intervention, grounded in self-determination theory, to enhance satisfaction, motivation, physical activity, and quality of life in adults with spinal cord injury (SCI) (*n* = 11) (85). Finally, an android application in a tablet together with a physiologic monitor was used as a telehealth system in 20 patients with PD to explore two different internet engagement trainings: a tele-coach assisted training (*n* = 10), and a self-regulated exercise training (*n* = 10) (78).

Other frequent strategies used for engagement in telerehabilitation are those directed to enhance patients' adherence and compliance to the therapy. Concerning this, in this systematic review, one study used a mobile web portal (wbPRO) to evaluate patient-reported outcomes in terms of feasibility, reliability, adherence, and subject-perceived benefits in 31 patients with MS, to quantify the impact of MS-related symptoms on the perceived patients' well-being (77). Moreover, a more sophisticated telerehabilitation system (SENSE-PARK system) including a set of wearable sensors (three to be used during the day and one at night), a Wii Balance Board software, and a smartphone application was used at patients' home to assess the feasibility and usability of the system, in 22 patients with PD (72). Further, a web-based physiotherapy platform with weekly personal, conversational support was used in patients with MS (*n* = 45), compared to a usual home paper format protocol (*n* = 45) to explore the user experience and feasibility of a web-based intervention (82).

Finally, in this systematic review, two studies directed to investigate the emotional components of the engagement strategies when using telerehabilitation systems were also found. These types of engagement strategies are embedded into the emotional dimension of engagement (88), usually implemented by using telephone and email interviews. Particularly, two studies were directed to enhance the emotional dimensions of engagement (76, 79). Specifically, in the study conducted by Houlihan et al., the therapists assessed the results obtained from a telephone-based health self-management intervention in patients with SCI (*n* = 42), compared with a usual care control group (*n* = 42). However, in the study conducted by Skolasky et al., the clinical staff involved in the study used motivational interviewing strategies to elicit and strengthen motivation for change in patients with MS (*n* = 31).

#### Engagement as a Secondary Outcome

Seven studies of this systematic review aimed to use telerehabilitation training for motor, cognitive, or logopedic interventions in patients with neurological disorders and to enhance patient engagement as a secondary outcome. Specifically, in this review, three studies were directed to investigate user experience, and system feasibility when using telerehabilitation systems for other neurorehabilitation proposes (73, 83, 86). As an example, in the study conducted by Ellis et al., they explored the preliminary effectiveness, safety, and acceptance of a mobile health (mHealth) application–a mediated exercise program– designed to promote sustained physical activity in 23 patients with PD. Moreover, in another study, the authors assessed the feasibility and potential clinical changes associated with telerehabilitation training for upper limb recovery, based in a robotic technology-supported arm, supported by a video-game training system in 24 patients with chronic stroke (73). Finally, De Vries et al. reported the opinion of 16 patients with PD when using a home-based system without video movement analysis (83).

Moreover, the other five studies aimed at investigating engagement as a secondary outcome when using telerehabilitation systems for neurorehabilitation proposes. Specifically, one study investigated changes in aphasia severity, communication-related quality of life, and participation, in 19 patients with aphasia while using the TeleGAIN telerehabilitation system (80). Moreover, another study investigated postural control and balance improvements after a 10-week of a virtual Kinect home-exercise program in 24 adults with MS, and assessed patients' adherence and motivation when using the telerehabilitation system as a secondary outcome (75). In one study conducted by Yeh et al., the authors tested a telerehabilitation system composed of two subsystems: a motor rehabilitation system and a telecommunication system to improve the mobility of patients with stroke and to motivate them to continue with the telerehabilitation training (69). Finally, in another study, the effectiveness of a virtual reality-based telerehabilitation program for balance recovery in chronic stroke patients was assessed and compared to the usual rehabilitation training (74).

### Engagement Assessment

Among the analyzed studies in this systematic review, the following main three assessment methods have been found to assess patient engagement: measurement scales, telephone based-interviews, and paper diaries. Regarding the measurement scales in the study conducted by Lloréns et al. (70), the authors used the Self-Awareness Deficits Interview (SADI) scale (89), and the Social Skills Scale (SSS) (90). However, others used the Short Form-36 (SF-36) (91), and the Hospital Anxiety Depression Scale (HADS) (92) to assess engagement as a secondary outcome (81). Moreover, the Communication Life Scale and the communicative activities checklist were used in patients with aphasia to assess engagement as a secondary outcome (80). Finally, three scales directed to assess engagement as a primary outcome were used. The Intrinsic Motivation Inventory (IMI) (93), was used to assess the level of motivation in patients with stroke after the telerehabilitation period (73). The Patients Activation Measure (PAM) (23), was used to assess health self-management in patients with SCI (76). Finally, the Profile of Mood States (POMS) questionnaire (94) was used in patients with SCI or ABI after the telerehabilitation training period (69). Table 5 aims to summarize the different scale measures, and the aim of each engagement scale measure.

**Table 5:** Summary of engagement scale measures.

**Engagement scale measures**	**Type**	**Aim**
Self-Awareness Deficits Interview (SADI) scale (89)	An interviewer-rated, semi-structured interview	To obtain both qualitative and quantitative data on the status of self-awareness following TBI. The interview has three areas of questions: (1) self-awareness of deficits; (2) self-awareness of functional implications of deficits; and (3) ability to set realistic goals
Intrinsic Motivation Inventory (IMI) (93)	Short- or long-form questionnaire	To measure grounded on the Self-Determination Theory (SDT) used in assessing the subjective experiences of participants when developing an activity. Specifically, it evaluates interest and enjoyment in a task, along with several other factors
Patients Activation Measure (PAM) (23)	A valid, highly reliable, unidimensional, probabilistic Guttman-like scale	To reflect a developmental model of activation, by assessing four different stages in patients activation: (1) believing the patient role is important, (2) having the confidence and knowledge necessary to take action, (3) taking action to maintain and improve one's health, and (4) staying the course even under stress
Profile Of Mood States (POMS) questionnaire (94)	A long (65 items) or short (35 items) questionnaires that contain a series of descriptive words/statements that describe feelings people have. The subjects self-report on each of these areas using a 5-point Likert scale	To measure peoples' mood state

### Engagement Outcomes

#### Engagement as a Primary Outcome

Regarding the outcomes observed in the analyzed studies which aimed to foster patient engagement as a primary outcome, we observed the following reported outcomes. The VR game proposed in the study conducted by Llorens et al., improved self-awareness and social cognition deficits in patients with ABI and PD after 8 months of a telerehabilitation training (70). Through a smartphone TeleCoach application, patients with MS increased activity and reduced fatigue levels after 12 weeks of training, improving patients' self-management (81). Moreover, another study demonstrated that by replicating rehabilitation group dynamics through a telerehabilitation system is possible to enhance patient engagement to the rehabilitation training in patients with MS (84). Regarding the use of telerehabilitation training in patients with stroke, one study showed that by using an iPad training stroke survivors experienced increased participation in therapeutic activities, increased socialization, as well as less inactivity and boredom (71). In addition to this, the results obtained in the study conducted by Nijenhuis et al. showed an increased motivation to participate in the rehabilitation training when using a remotely monitored training system at home (73). However, in another study conducted in patients with PD, the patients reported that direct feedback about the patients' health condition when using the telerehabilitation training system would help to increase patients' motivation (72). Another study showed that patients with PD benefit from a mobile biofeedback system that provides real feedback about patients' health conditions, and enhance patient engagement to the rehabilitation routine (86). Furthermore, in one study in which patients with stroke could feeling the sense of the co-presence of the therapist during the telerehabilitation training, the psychological state of the patients was improved (69). However, in contrast to the above-commented studies, one study reported a reduction in patients' self-efficacy and willingness regardless of patients' fatigue after the telerehabilitation training (69).

Finally, one study highlighted the importance of building in conversations by weekly interviews with people with MS about expectations of exercise and its potential benefits, particularly with those patients whose physical and mental conditions may be deteriorating while using motor telerehabilitation systems (82). In this regard, another study reported that health behavior change counseling by telephone-based interventions could improve health outcomes during the first 12 months after the surgical procedure in patients operated of spinal stenosis, improving patient engagement to the rehabilitation program (79). Moreover, 6 months of a telerehabilitation period based in a telephonic intervention program showed a more significant change in PAM scores, as well as a higher decrease in social/role activity limitations, and improvements in services/resources awareness in patients with SCI (76). Further, another telerehabilitation training using an online video-chat platform increase autonomous motivation in patients with SCI (85).

#### Engagement as a Secondary Outcome

Regarding the outcomes observed in the analyzed studies which aimed to foster patient engagement as a secondary outcome, we observed the following reported outcomes. One study reported improvements in communication-related quality of life in patients with aphasia, and a decrease of the aphasia severity, which lead to an increase of patient engagement in communicative activities (80). Another study conducted by Palacios-Ceña et al. highlighted the following positive factors reported by patients with MS after using a Kinect telerehabilitation systems: (1) the Kinect training increased the level of independence of the patients; (2) the patients reported to can share their illness state with their relatives'; (3) the patients reported positive effects about the incorporation of a videogame for rehabilitation, and (4) the patients reported positive effects regarding the possibility of evaluating themselves through the feedback provided by the telerehabilitation system (75).

#### Engagement Strategies Effectiveness

Overall, we found different patient engagement strategies throughout the 18 analyzed studies. Table 6 summarizes the different engagement strategies found among the analyzed studies, and the level of effectiveness of such engagement strategies for teleneurorehabilitation (positive, neutral, or negative). Specifically, 12 studies reported positive results when using tele-neurorehabilitation interventions for patient engagement (69, 70, 73, 75, 76, 78–83, 85). Five studies reported neutral effects in patient engagement after the tele-neurorehabilitation training period (71, 72, 74, 84, 86). Finally, only one study out of the 18 analyzed studies reported negative results in patients' adherence to the training after the telerehabilitation training period (77).

**Table 6:** Summary of engagement variables in tele-neurorehabilitation and engagement improvement.

**Included studies**	**Self-awareness/ Self-management**	**Adherence to the intervention/Satisfaction**	**Emotional support**	**Patient activation/motivation**	**Engagement improvement**
Yeh et al. (69)		X	X		Positive
Lloréns et al. (70)	X				Positive
White et al. (71)		X	X	X	Neutral
Ferreira et al. (72)		X		X	Neutral
Nijenhuis et al. (73)		X		X	Positive
Lloréns et al. (74)		X		X	Neutral
Palacios-Ceña et al. (75)		X		X	Positive
Houlihan et al. (76)	X	X	X	X	Positive
Engelhard et al. (77)	X	X	X		Negative
D'hooghe et al. (81)	X	X	X		Positive
Lai et al. (78)	X	X			Positive
Skolasky et al. (79)		X	X		Positive
Pitt et al. (80)		X		X	Positive
De Vries et al. (83)		X		X	Positive
Dennett et al. (82)		X	X		Positive
Thomas et al. (84)	X	X	X		Neutral
Chemtob et al. (85)		X	X	X	Positive
Ellis et al. (86)		X			Neutral

## Discussion

The engagement of patients in the rehabilitation process is considered a primary aim for worldwide healthcare interventions [see (95)]. Patient engagement is considered a key component in neurorehabilitation in order to promote greater neuroplastic changes and functional outcomes (2). In this concern, digital technologies have been considered as a useful resource for enhancing patients' participation, allowing them to have an active role in their healthcare process (96, 97). The introduction of digital technologies in the field of neurorehabilitation has prompted the possibility to conduct the rehabilitation protocol at patients' homes (16, 98). Thus, telerehabilitation protocols save time for the patient by reducing displacements to the hospital, and the clinicians can follow the patients after the hospital discharge from the hospital (16, 98). However, which is the role of engagement when using tele-rehabilitation systems in neurorehabilitation? The here presented systematic review aims at reviewing the different engagement strategies and different engagement assessments while using telerehabilitation systems for neurorehabilitation.

In this systematic review, the studies were first divided into those in which patients' engagement was considered a first outcome of the telerehabilitation training, and those in which engagement was considered a secondary outcome of the telerehabilitation training. Interestingly, more studies that considered patients engagement as a primary outcome of the telerehabilitation training (*N* = 11), compared to those that considered patients engagement as a secondary outcome (*N* = 7) were found. Particularly, most of the analyzed studies that were directed to enhance patients' engagement through telerehabilitation systems in neurorehabilitation, had been conducted during the last 4 years from 2015 to 2019 (70–72, 76–79, 81, 82, 84, 85). This data indicates that fostering patients' engagement through the use of new technologies in neurorehabilitation has been a matter of interest for several years. Interestingly, this data is in line with the systematic review conducted by Barello et al. (99), in which they looked for studies using e-Health interventions for patient engagement, and highlighted the necessity of conducting more studies investigating the use of new digital technologies to enhance patient engagement. The data collected in this systematic review confirms that there was a progressive increase in the use of new technologies to engage patients, specifically those with neurological disorders, into their rehabilitation process. Secondly, our results showed an increase in interest in creating new telerehabilitation protocols in neurorehabilitation for enhancing patients' engagement by promoting patients' self-awareness and self-management (*N* = 6), patients' motivation (*N* = 9), and emotional support (*N* = 9). Such engagement components have been described as components of the behavioral and cognitive dimension of patients' engagement (30). Thus, in this systematic review, the studies analyzed were directed at fostering the behavioral and cognitive dimension through the use of telerehabilitation systems in patients with neurological diseases. These findings are supported by other investigations that were also directed at fostering the behavioral and cognitive dimension of engagement during the rehabilitation process of different clinical populations (100, 101). Concerning this, the results of this systematic review show that the use of telerehabilitation systems in patients with neurological disorders are useful for fostering the behavioral and cognitive dimension of engagement and for increase patients engagement with the rehabilitation program (73, 77, 78, 81, 84, 86). One explanation of this could be that through the telerehabilitation systems it is possible to give a real feedback to the patients about their physical and physiological conditions, as well as the possibility to interact with the telerehabilitation system (70, 73–75, 78, 81, 83). Concerning this, the studies of this systematic review are consistent with later investigations that demonstrated the effectiveness of digital technologies in inducing behavioral, physiological, and emotional responses by giving an immediate real feedback about such responses to the patients (22, 102–104). Moreover, such investigations were also directed at fostering the emotional dimension of the engagement, referring to the patients' acceptance of the disease, to an adequate adjustment to their illness (105), and improving the quality of the relationship between clinicians and patients (24). Specifically, in the analyzed studies of this systematic review, the emotional dimension of engagement has been tackled by using weekly telephonic interviews (72, 76, 84), using a face to face communication through on-line digital platforms (78, 80, 85), or by giving positive and motivating messages to the patients during the telerehabilitation training (78, 81).

Regarding the assessment of engagement during the telerehabilitation training in neurorehabilitation, the studies analyzed in this systematic review show that, at the moment, there are few available scales to assess the level of patient engagement and to deeply assess the different components of engagement. However, some available measures providing quantitative data about patient engagement such as the PAM (23), IMI (93), and the SADI (89), and POMS questionnaire (94) scales are available. Out of these four measures scales, the newest and the most used one is the PAM, which, as described in Table 5, enables the assessment of the patient activation during their healthcare routine in-depth. Although the PAM seems one of better measures to assess patient engagement, the POMS questionnaire could be an excellent complement to further assess the emotional state of the patients in their daily healthcare routine and during the telerehabilitation period in patients with neurological disease. The SADI is limited to patients with traumatic brain injury, and this limits the use of this scale to assess self-awareness of the illness in patients with other neurological pathologies. Finally, the IMI could be replaced by the PAM, as this is the newest measure that contemplates more aspects of patient activation in comparison to the IMI. Further, the results obtained in the PAM can reflect patient motivation to participate in their healthcare routine. Besides the quantitative engagement measures, a significant amount of studies that use interviews and diary reports for the qualitative assessment of patient engagement when using telerehabilitation systems were found. In this regard, it is known that data from motivational interviews play an essential role in evaluating patient engagement during the rehabilitation period (106, 107). Moreover, the efficacy of using semi-structured interviews to foster patients with chronic illness to participate in their healthcare routine has been demonstrated (108).

Finally, regarding the effectiveness of the engagement strategies used in the analyzed studies of this systematic review, 12 studies out of 18 reported positive outcomes in fostering patient engagement after the telerehabilitation training. In particular, the engagement strategies used in these 12 studies were mainly focused on patient participation, patient decision making, and patient self-management, all of them involved in the behavioral, cognitive, and emotional dimensions of engagement (see Table 6). Such positive results are in line with later studies in which a motivational model to foster participation in the neurorehabilitation programs was proposed (109). Moreover, others also proposed new neurorehabilitation strategies by enhancing patient self-management, self-awareness, and motivation in rehabilitation routines (2). Most of the revised studies in this systematic review presented positive results by enhancing the behavioral, cognitive, and emotional dimensions of patient engagement. However, most of them used a “monomethod” study design, directed at assessing qualitative or quantitative engagement outcomes.

## Limitations

The present systematic review shows the following limitations regarding the standard protocols for systematic reviews: no registration in a public database, a librarian was not included in the bibliographic research stage, and no duplicate and independent searches of the studies were done.

## Conclusions

The studies commented throughout this systematic review pave the way for the design of new telerehabilitation protocols, not only focusing on measuring quantitative or qualitative measures but measuring both of them through a mixed model intervention design (1). The future clinical studies with a mixed model design will provide more abundant data regarding the role of engagement in telerehabilitation, leading to a possibly greater understanding of its underlying components.

## Data Availability Statement

All datasets generated for this study are included in the article/supplementary material.

## Author Contributions

MM-G and OR developed the paper concept. MM-G carried out the bibliographic review, was responsible for the methodology, and wrote the manuscript draft. MM and JM contributed to the drafting of the manuscript. FB, FR, and PM gave bibliographic suggestions and reviewed the manuscript for important intellectual content. GR, FM, and OR supervised the editing and revisions for important intellectual content. All the authors approved the final version of the manuscript for submission.

## Conflict of Interest

The authors declare that the research was conducted in the absence of any commercial or financial relationships that could be construed as a potential conflict of interest.

## References

[1] Powell H Mihalas S Onwuegbuzie AJ Suldo S Daley CE Mixed methods research in school psychology: a mixed methods investigation of trends in the literature. Psychol. Sch. (2008) 45:291–309. 10.1002/pits.20296

[2] Danzl MM Etter NM Andreatta RO Kitzman PH. Facilitating neurorehabilitation through principles of engagement. J Allied Health. (2012) 41:35–41. 22544406

[3] Kleim JA Jones TA. Principles of experience-dependent neural plasticity: implications for rehabilitation after brain damage. J Speech Lang Hear Res. (2008) 51:S225–39. 10.1044/1092-4388(2008/018) 18230848

[4] Phillips C. Lifestyle modulators of neuroplasticity: how physical activity, mental engagement, and diet promote cognitive health during aging. Neural Plast. (2017) 2017:3589271. 10.1155/2017/3589271 28695017 PMC5485368

[5] Moucha R Kilgard MP Chapter 7 cortical plasticity and rehabilitation. Progress in Brain Research. Elsevier (2006) 157:111–389. 10.1016/S0079-6123(06)57007-4 17167905

[6] Warraich Z Kleim JA. Neural plasticity: the biological substrate for neurorehabilitation. PMandR. (2010) 2:S208–19. 10.1016/j.pmrj.2010.10.016 21172683

[7] Matthews G Warm JS Reinerman LE Langheim LK Saxby DJ Task engagement, attention, and executive control. In: Gruszka A Matthews G Szymura B editors. Handbook of Individual Differences in Cognition. The Springer Series on Human Exceptionality. New York, NY: Springer (2010). p. 205–30.

[8] Nadeau SE. A paradigm shift in neurorehabilitation. Lancet Neurol. (2002) 1:126–30. 10.1016/S1474-4422(02)00044-3 12849517

[9] Bovend'eerdt TJH Dawes H SackleyC Wade DT. Practical research-based guidance for motor imagery practice in neurorehabilitation. Disabil Rehabil. (2012) 34:2192–200. 10.3109/09638288.2012.676703 22533623

[10] Miller KL. Patient centered care: a path to better health outcomes through engagement and activation. NeuroRehabilitation. (2016) 39:465–70. 10.3233/NRE-161378 27689606

[11] Lupton D Digital Health: Critical and Cross-Disciplinary Perspectives. Critical Approaches to Health. New York, NY: Routledge/Taylor and Francis Group (2018). Available online at: https://search.proquest.com/docview/2009246760?accountid=16562

[12] Foster CH Richards J Thorogood M Hillsdon M Kaur A Wickramasinghe KK Remote and Web 2.0 interventions for promoting physical activity. Cochrane Database Syst Rev. (2013) CD010395 10.1002/14651858.CD010395 PMC9674455 24085594

[13] Serino S Baglio F Rossetto F Realdon O Cipresso P Parsons TD. Picture Interpretation Test (PIT) 360°: an innovative measure of executive functions. Sci Rep. (2017) 7:16000. 10.1038/s41598-017-16121-x 29167494 PMC5700040

[14] Realdon O Serino S Savazzi F Rossetto F Cipresso P Parsons TD. An ecological measure to screen executive functioning in MS: the Picture Interpretation Test (PIT) 360°. Sci Rep. (2019) 9:5690. 10.1038/s41598-019-42201-1 30952936 PMC6450934

[15] Piron L Turolla A Agostini M Zucconi C Cortese F Zampolini M. Exercises for paretic upper limb after stroke: a combined virtual-reality and telemedicine approach. J Rehabil Med. (2009) 41:1016–20. 10.2340/16501977-0459 19841835

[16] Agostini M Lorenzo M Rita B Vanna P Paolo T Annalena V. Telerehabilitation and recovery of motor function: a systematic review and meta-analysis. J Telemed Telecare. (2015) 21:202–13. 10.1177/1357633X15572201 25712109

[17] Tella SD Pagliari Blasi V Mendozzi L Rovaris M Baglio F. Integrated telerehabilitation approach in multiple sclerosis: a systematic review and meta-analysis. J Telemed Telecare. (2019) 1–15. 10.1177/1357633X19850381 31132909

[18] Isernia S Pagliari C Jonsdottir J Castiglioni C Gindri P Gramigna C. Efficiency and patient-reported outcome measures from clinic to home: the human empowerment aging and disability program for digital-health rehabilitation. Front Neurol. (2019) 10:1206. 10.3389/fneur.2019.01206 31824398 PMC6882300

[19] Realdon O Rossetto F Nalin M Baroni I Cabinio M Fioravanti R. Technology-enhanced multi-domain at home continuum of care program with respect to usual care for people with cognitive impairment: the ability-telerehabilitation study protocol for a randomized controlled trial. BMC Psychiatry. (2016) 16:425. 10.1186/s12888-016-1132-y 27887597 PMC5123349

[20] Palsbo SE Bauer D. Telerehabilitation: Managed Care's New Opportunity. Managed Care Quarterly (2000). 11146846

[21] Bensing J. Bridging the gap: the separate worlds of evidence-based medicine and patient-centered medicine. Pat Educ Couns. (2000) 39:17–25. 10.1016/S0738-3991(99)00087-7 11013544

[22] Graffigna G Barello S Riva G Bosio AC. Patient engagement: the key to redesign the exchange between the demand and supply for healthcare in the era of active ageing. Stud Health Technol Inform. (2014) 203:85–95. 10.3233/978-1-61499-425-1-85 26630515

[23] Hibbard JH Stockard J Mahoney ER Tusler M. Development of the Patient Activation Measure (PAM): conceptualizing and measuring activation in patients and consumers. Health Serv Res. (2004) 39:1005–26. 10.1111/j.1475-6773.2004.00269.x 15230939 PMC1361049

[24] Graffigna G Barello S Innovating healthcare in the era of patient engagement: challenges, opportunities and new trends. In: Patient Engagement: A Consumer-Centered Model to Innovate Healthcare. Berlin, Boston: De Gruyter (2016). p. 1–12. 10.1515/9783110452440

[25] Fisher ES McClellan MB Safran DG. Building the path to accountable care. N Engl J Med. (2011) 365:2445–7. 10.1056/NEJMp1112442 22204720

[26] Entwistle VA Watt IS. Patient involvement in treatment decision-making: the case for a broader conceptual framework. Pat Educ Couns. (2006) 63:268–78. 10.1016/j.pec.2006.05.002 16875797

[27] Marteau TM Dormandy E. Facilitating informed choice in prenatal testing: how well are we doing? Am J Med Genet. (2001) 106:185–90. 10.1002/ajmg.10006 11778978

[28] Charles C Gafni A Whelan T. How to improve communication between doctors and patients. Br Med J. (2000) 320:1220. 10.1136/bmj.320.7244.1220 10797016 PMC1117979

[29] Charles C Gafni A Whelan T. Decision-making in the physician-patient encounter: revisiting the shared treatment decision-making model. Soc Sci Med. (1999) 49:651–61. 10.1016/S0277-9536(99)00145-8 10452420

[30] Graffigna G Barello S Triberti S Giving (Back) a role to patients in the delivery of healthcare services: theoretical roots of patient engagement. In: Patient Engagement: A Consumer-Centered Model to Innovate Healthcare. Berlin, Boston, MA: De Gruyter (2016). p. 13–26.

[31] Barbosa CD Balp MM Kulich K Germain N Rofail D. A literature review to explore the link between treatment satisfaction and adherence, compliance, and persistence. Pat Prefer Adher. (2012) 6:38–48. 10.2147/PPA.S24752 22272068 PMC3262489

[32] Ingram TL. Compliance: a concept analysis. Nursing Forum. (2009) 44:189–94. 10.1111/j.1744-6198.2009.00142.x 19691655

[33] Stewart M Towards a global definition of patient centred care: the patient should be the judge of patient centred care. Br Med J. (2001) 322:444–5. 10.1136/bmj.322.7284.444 11222407 PMC1119673

[34] Barlow J. How to use education as an intervention in osteoarthritis. Best Pract Res Clin Rheumatol. (2001) 15:545–58. 10.1053/berh.2001.0172 11567538

[35] Barlow J Wright C Sheasby J Turner A Hainsworth J. Self-management approaches for people with chronic conditions: a review. Pat Educ Couns. (2002) 48:177–87. 10.1016/S0738-3991(02)00032-0 12401421

[36] Aujoulat I d'Hoore W Deccache A Patient empowerment in theory and practice: polysemy or cacophony?” Pat Educ Couns. (2007) 66:13–20. 10.1016/j.pec.2006.09.008 17084059

[37] Feste C Anderson RM. Empowerment: from philosophy to practice. Pat Educ Couns. (1995) 26:139–44. 10.1016/0738-3991(95)00730-N 7494713

[38] Anderson RM Martha MF. Patient empowerment: myths and misconceptions. Pat Educ Couns. (2010) 79:277–82. 10.1016/j.pec.2009.07.025 19682830 PMC2879465

[39] Greene J Hibbard JH. Why does patient activation matter? An examination of the relationships between patient activation and health-related outcomes. J Gen Intern Med. (2012) 27:520–6. 10.1007/s11606-011-1931-2 22127797 PMC3326094

[40] Greene J Hibbard JH Tusler M How Much Do Health Literacy and Patient Activation Contribute to Older Adults' Ability to Manage Their Health? AARP Public Policy Institute (2005).

[41] Graffigna G Barello S Bonanomi A Lozza E. Measuring patient engagement: development and psychometric properties of the Patient Health Engagement (PHE) scale. Front Psychol. (2015). 6:274. 10.3389/fpsyg.2015.00274 25870566 PMC4376060

[42] Riva G Baños RM Botella C Wiederhold BK Gaggioli A. Positive technology: using interactive technologies to promote positive functioning. Cyberpsychol Behav Soc Netw. (2012) 15:69–77. 10.1089/cyber.2011.0139 22149077

[43] Triberti S Riva G Positive technology for enhancing the patient engagement experiences. Patient Engagement: A Consumer-Centered Model to Innovate Healthcare. Berlin; Boston, MA: De Gruyter (2016). 10.1515/9783110452440-005

[44] Seligman MEP Positive psychology, positive prevention, and positive therapy. Handbook of Positive Psychology (2002). p. 3–12.

[45] Argenton L Triberti S Serino S Muzio M Riva G Serious games as positive technologies for individual and group flourishing. Stud. Comput. Intell. (2014) 536:221–244. 10.1007/978-3-642-45432-5_11

[46] Frome J Eight ways videogames generate emotion. In: 3rd Digital Games Research Association International Conference: “Situated Play”, DiGRA 2007. Tokyo (2007).

[47] Tachakra S Wang XH Istepanian RSH Song YH. Mobile E-health: the unwired evolution of telemedicine. Telemed J E-Health. (2003) 9:247–57. 10.1089/153056203322502632 14611692

[48] Sinclair P Kable A Levett-Jones T. The effectiveness of internet-based e-learning on clinician behavior and patient outcomes: a systematic review protocol. JBI Database Syst Rev Implement Rep. (2015) 13:52–64. 10.11124/jbisrir-2015-1919 26447007

[49] Giggins OM Persson UMC Caulfield B. Biofeedback in rehabilitation. J Neuroeng Rehabil. (2013) 10:60. 10.1186/1743-0003-10-60 23777436 PMC3687555

[50] Repetto C Gorini A Algeri D Vigna C Gaggioli A Riva G The use of biofeedback in clinical virtual reality: the intrepid project. Stud Health Technol Inform. (2009) 7:128–32. 10.3791/1554 19592748

[51] Turolla A Dam M Ventura L Tonin P Agostini M Zucconi C. Virtual reality for the rehabilitation of the upper limb motor function after stroke: a prospective controlled trial. J Neuroeng Rehabil. (2013) 10:85. 10.1186/1743-0003-10-85 23914733 PMC3734026

[52] Salisbury DB Dahdah M Driver S Parsons TD Richter KM. Virtual reality and brain computer interface in neurorehabilitation. Baylor Univ Med Center Proc. (2017) 29:124–7. 10.1080/08998280.2016.11929386 27034541 PMC4790543

[53] Rinne P Mace M Nakornchai T Zimmerman K Fayer S Sharma P. Democratizing neurorehabilitation:how accessible are low-cost mobile-gaming technologies for self-rehabilitation of arm disability in stroke? PLoS ONE. (2016) 11:413. 10.1371/journal.pone.0163413 27706248 PMC5051962

[54] Rogante M Grigioni M Cordella D Giacomozzi C. Ten years of telerehabilitation: a literature overview of technologies and clinical applications. NeuroRehabilitation. (2010) 27:287–304. 10.3233/NRE-2010-0612 21160118

[55] Barnes MP Radermacher H. Neurological rehabilitation in the community. J Rehabil Med. (2001) 33:244–8. 10.1080/165019701753236419 11766952

[56] Atwal A Kirsty T Kay C Christine C. Multidisciplinary perceptions of the role of nurses and healthcare assistants in rehabilitation of older adults in acute health care. J Clin Nurs. (2006) 15:1418–25. 10.1111/j.1365-2702.2005.01451.x 17038103

[57] Hailey D Roine R Ohinmaa A Dennett L. Evidence of benefit from telerehabilitation in routine care: a systematic review. J Telemed Telecare. (2011) 17:281–7. 10.1258/jtt.2011.101208 21844172

[58] Huijgen BC Vollenbroek-Hutten MMR Zampolini M Opisso E Bernabeu M van Nieuwenhoven J J . Feasibility of a home-based telerehabilitation system compared to usual care: arm/hand function in patients with stroke, traumatic brain injury and multiple sclerosis. J Telemed Telecare. (2008) 14:249–56. 10.1258/jtt.2008.080104 18633000

[59] Chen J Liu M Sun D Jin Y Wang T Ren C. Effectiveness and neural mechanisms of home-based telerehabilitation in patients with stroke based on FMRI and DTI: a study protocol for a randomized controlled trial. Medicine. (2018) 97:e9605. 10.1097/MD.0000000000009605 29504985 PMC5779754

[60] Piron L Tonin P Trivello E Battistin L Dam M. Motor tele-rehabilitation in post-stroke patients. Inform Health Soc Care. (2004) 29:119–25. 10.1080/14639230410001723428 15370992

[61] Chumbler NR Quigley P Li X Morey M Rose D Sanford J. Effects of telerehabilitation on physical function and disability for stroke patients: a randomized, controlled trial. Stroke. (2012) 43:2168–74. 10.1161/STROKEAHA.111.646943 22627983

[62] Giansanti D Macellari V Maccioni G. Telemonitoring and telerehabilitation of patients with Parkinson's disease: health technology assessment of a novel wearable step counter. Telemed e-Health. (2008) 14:76–83. 10.1089/tmj.2007.0019 18328028

[63] Gandolfi M Geroin C Dimitrova E Boldrini P Waldner A Bonadiman S. Virtual reality telerehabilitation for postural instability in Parkinson's disease: a multicenter, single-blind, randomized, controlled trial. Biomed Res Int. (2017) 2017:7962826. 10.1155/2017/7962826 29333454 PMC5733154

[64] Khan F Amatya B Kesselring J Galea M. Telerehabilitation for persons with multiple sclerosis. Cochrane Database Syst Rev. (2015) 4:CD010508. 10.1002/14651858.CD010508.pub2 25854331 PMC7211044

[65] Liberati A Altman DG Tetzlaff J Mulrow C Gøtzsche PC Ioannidis JPA The PRISMA statement for reporting systematic reviews and meta-analyses of studies that evaluate health care interventions: explanation and elaboration. J Clin Epidemiol. (2009) 64:e1–34. 10.1016/j.jclinepi.2009.06.006 19631507

[66] Schardt C Adams MB Owens T Keitz S Fontelo P. Utilization of the PICO framework to improve searching pubmed for clinical questions. BMC Med Informat Decis Mak. (2007) 7:16. 10.1186/1472-6947-7-16 17573961 PMC1904193

[67] Elmagarmid A Fedorowicz Z Hammady H Ilyas I Khabsa M Ouzzani M Rayyan: a systematic reviews web app for exploring and filtering searches for eligible studies for cochrane reviews. In: Evidence-Informed Public Health: Opportunities and Challenges. Abstracts of the 22nd Cochrane Colloquium. Hyderabad: John Wiley & Sons (2014).

[68] Sterne JAC Savovi? J Page MJ Elbers RG Blencowe NS Boutron I. RoB 2: a revised tool for assessing risk of bias in randomised trials. BMJ. (2019) 366:14898. 10.1136/bmj.l4898 31462531

[69] Yeh SC McLaughlin M Nam Y Sanders S Chang C Kennedy B Emotions and telerebabilitation: pilot clinical trials for virtual telerebabilitation application using haptic device and its impact on post stroke patients' mood and motivation. In: Shumaker R editor. International Conference on Virtual and Mixed Reality. VMR 2011: Virtual and Mixed Reality - Systems and Applications. Berlin; Heidelberg: Springer (2011). p. 119–128.

[70] Lloréns R Navarro MD Alcañiz M Noé E Therapeutic effectiveness of a virtual reality game in self-awareness after acquired brain injury. Ann Rev CyberTher Telemed. (2012) 10:297–301. 10.3233/978-1-61499-121-2-297 22954875

[71] White JH Janssen H Jordan L Pollack M. Tablet technology during stroke recovery: a survivor's perspective. Disabil Rehabil. (2015) 37:1186–92. 10.3109/09638288.2014.958620 25212736

[72] Ferreira JJ Godinho C Santos AT Domingos J Abreu D Lobo R. Quantitative home-based assessment of parkinson's symptoms: the SENSE-PARK feasibility and usability study. BMC Neurol. (2015) 15:89. 10.1186/s12883-015-0343-z 26059091 PMC4460963

[73] Nijenhuis SM Prange GB Amirabdollahian F Sale P Infarinato F Nasr N. Feasibility study into self-administered training at home using an arm and hand device with motivational gaming environment in chronic stroke. J Neuroeng Rehabil. (2015) 12:89. 10.1186/s12984-015-0080-y 26452749 PMC4599772

[74] Lloréns R Noé E Colomer C Alcañiz M. Effectiveness, usability, and cost-benefit of a virtual reality-based telerehabilitation program for balance recovery after stroke: a randomized controlled trial. Arch Phys Med Rehabil. (2015) 96:418–25.e2. 10.1016/j.apmr.2014.10.019 25448245

[75] Palacios-Ceña D Ortiz-Gutierrez RM Buesa-Estellez A Galân-Del-Rio F Cachón-Pérez JM Martinez-Piedrola R. Multiple sclerosis patients' experiences in relation to the impact of the kinect virtual home-exercise programme: a qualitative study. Eur J Phys Rehabil Med. (2016) 52:347–55. 26883340

[76] Houlihan BV Brody M Everhart-Skeels S Pernigotti D Burnett S Zazula J. Randomized trial of a peer-led, telephone-based empowerment intervention for persons with chronic spinal cord injury improves health self-management. Arch Phys Med Rehabil. (2017) 98:1067–76.e1. 10.1016/j.apmr.2017.02.005 28284835

[77] Engelhard MM Patek SD Sheridan K Lach JC Goldman MD. Remotely engaged: lessons from remote monitoring in multiple sclerosis. Int J Med Inform. (2017) 100:26–31. 10.1016/j.ijmedinf.2017.01.006 28241935 PMC5331862

[78] Lai B Bond K Kim Y Barstow B Jovanov E Bickel CS. Exploring the uptake and implementation of tele-monitored home-exercise programmes in adults with Parkinson's disease: a mixed-methods pilot study. J Telemed Telecare. (2018) 26:53–63. 10.1177/1357633X18794315 30134777

[79] Skolasky RL Maggard AM Wegener ST Riley LH. Telephone-based intervention to improve rehabilitation engagement after spinal stenosis surgery: a prospective lagged controlled trial. J Bone Joint Surg Am Vol. (2018) 100:21–30. 10.2106/JBJS.17.00418 29298257 PMC6153441

[80] Pitt R Theodoros D Hill AJ Russell T. The impact of the telerehabilitation group aphasia intervention and networking programme on communication, participation, and quality of life in people with aphasia. Int J Speech Lang Pathol. (2018) 21:1–11. 10.1080/17549507.2018.1488990 30200788

[81] D'hooghe M Van Gassen G Kos D Bouquiaux O Cambron M Decoo D. Improving fatigue in multiple sclerosis by smartphone-supported energy management: the MS TeleCoach Feasibility Study. Mult Scler Relat Disord. (2018) 22:90–6. 10.1016/j.msard.2018.03.020 29649789

[82] Dennett R Coulter E Paul L Freeman J. A qualitative exploration of the participants' experience of a web-based physiotherapy program for people with multiple sclerosis: does it impact on the ability to increase and sustain engagement in physical activity? Disabil Rehabil. (2019) 1–8. 10.1080/09638288.2019.1582717 30907159

[83] De Vries NM Smilowska K Hummelink J Abramiuc B Van Gilst MM Bloem BR. Exploring the Parkinson patients' perspective on home-based video recording for movement analysis: a qualitative study. BMC Neurol. (2019) 19:71. 10.1186/s12883-019-1301-y 31029123 PMC6486968

[84] Thomas S Pulman A Thomas P Collard S Jiang N Dogan H. Digitizing a face-to-face group fatigue management program: exploring the views of people with multiple sclerosis and health care professionals via consultation groups and interviews. JMIR Form Res. (2019) 3:e10951. 10.2196/10951 31120021 PMC6549474

[85] Chemtob K Rocchi M Arbour-Nicitopoulos K Kairy D Fillion B Sweet SN Using tele-health to enhance motivation, leisure time physical activity, and quality of life in adults with spinal cord injury: a self-determination theory-based pilot randomized control trial. Psychol Sport Exerc. (2019) 43:243–52. 10.1016/j.psychsport.2019.03.008

[86] Ellis TD Cavanaugh JT DeAngelis T Hendron K Thomas CA Saint-Hilaire M. Comparative effectiveness of mhealth-supported exercise compared with exercise alone for people with parkinson disease: randomized controlled pilot study. Phys Ther. (2019) 99:203–16. 10.1093/ptj/pzy131 30715489

[87] Hoffmann TC Glasziou PP Boutron I Milne R Perera R Moher D. Better reporting of interventions: Template for Intervention Description and Replication (TIDieR) checklist and guide. BMJ. (2014) 348:g1687. 10.1136/bmj.g1687 24609605

[88] Graffigna G Barello S Riva G. Technologies for patient engagement. Health Aff. (2013) 32:1172. 10.1377/hlthaff.2013.0279 23733998

[89] Fleming JM Strong J Ashton R. Self-awareness of deficits in adults with traumatic brain injury: how best to measure? Brain Injury. (1996) 10:1–15. 10.1080/026990596124674 8680388

[90] Riggio RE Assessment of basic social skills. J Pers Soc Psychol. (1986) 51:649–60. 10.1037/0022-3514.51.3.649

[91] Stewart M. The medical outcomes study 36-item short-form health survey (SF-36). Aust J Physiother. (2007) 53:208. 10.1016/S0004-9514(07)70033-8 17899676

[92] Zigmond AS Snaith RP. The hospital anxiety and depression scale. Acta Psychiatr Scand. (1983) 67:361–70. 10.1111/j.1600-0447.1983.tb09716.x 6880820

[93] Ryan R Deci E Intrinsic Motivation Inventory (IMI) [Measurement Instrument]. The Intrinsic Motivation Inventory, Scale Description (1994). Available online at: http://selfdeterminationtheory.org/intrinsic-motivation-inventory (accessed January 01, 2018).

[94] McNair DM Lorr M Droppleman LF Manual for the POMS. Educational and Industrial Testing Service (1971). p. 205–30.

[95] Hardyman W Daunt KL Kitchener M Value co-creation through patient engagement in health care: a micro-level approach and research agenda. Public Manage Rev. (2015) 17:90–107. 10.1080/14719037.2014.881539

[96] Maye F Cox M Digital revolution – implementation of an electronic assistive technology pilot project in a neuro-rehabilitation setting. Int J Integr Care. (2017) 17:A112 10.5334/ijic.3417

[97] O'Neil O Fernandez MM Herzog J Beorchia M Gower V Gramatica F. Virtual reality for neurorehabilitation: insights from 3 European clinics. PM R. (2018) S198–206. 10.1016/j.pmrj.2018.08.375 30121365

[98] Perry JC Ruiz-Ruano JA Keller T. Telerehabilitation: toward a cost-efficient platform for post-stroke neurorehabilitation. In” IEEE International Conference on Rehabilitation Robotics, Zurich (2011). 10.1109/ICORR.2011.5975413 22275616

[99] Barello S Triberti S Graffigna G Libreri C Serino S Hibbard J. EHealth for patient engagement: a systematic review. Front Psychol. (2015) 6:2013. 10.3389/fpsyg.2015.02013 26779108 PMC4705444

[100] Sharry J Davidson R McLoughlin O Doherty G. A service-based evaluation of a therapist-supported online cognitive behavioral therapy program for depression. J Med Internet Res. (2013) 15:e121. 10.2196/jmir.2248 23807565 PMC3713925

[101] Robertson L Smith M Castle D Tannenbaum D. Using the internet to enhance the treatment of depression. Aust Psychiatry. (2006) 14:413–7. 10.1111/j.1440-1665.2006.02315.x 17116083

[102] Riva G Is presence a technology issue? Some insights from cognitive sciences. Virtual Real. (2009) 13:159–69. 10.1007/s10055-009-0121-6

[103] Gaggioli A Raspelli S Grassi A Pallavicini F Cipresso P Wiederhold BK. Ubiquitous health in practice: the interreality paradigm. Studies in Health Technology and Informatics. In MMVR (2011). p. 185–191. 21335786

[104] Serino S Triberti S Villani D Cipresso P Gaggioli A Riva G Toward a validation of cyber-interventions for stress disorders based on stress inoculation training: a systematic review. Virtual Real. (2014) 18:73–87. 10.1007/s10055-013-0237-6

[105] Barello S Graffigna G. Engaging patients to recover life projectuality: an Italian cross-disease framework. Qual Life Res. (2015) 24:1087–96. 10.1007/s11136-014-0846-x 25373927

[106] Burke BL Arkowitz H Menchola M. The efficacy of motivational interviewing: a meta-analysis of controlled clinical trials. J Consult Clin Psychol. (2003) 71:843–61. 10.1037/0022-006X.71.5.843 14516234

[107] Öhman A. Qualitative methodology for rehabilitation research. J Rehabil Med. (2005) 37:273–80. 10.1080/16501970510040056 16208859

[108] Pomey MP Ghadiri DP Karazivan P Fernandez N Clavel N. Patients as partners: a qualitative study of patients' engagement in their health care. PLoS ONE. (2015) 10:e0122499. 10.1371/journal.pone.0122499 25856569 PMC4391791

[109] Medley AR Powell T. Motivational interviewing to promote self-awareness and engagement in rehabilitation following acquired brain injury: a conceptual review. Neuropsychol Rehabil. (2010) 20:481–508. 10.1080/09602010903529610 20182952

